# The influence of pelvic tilt on stress distribution in the acetabulum: finite element analysis

**DOI:** 10.1186/s12891-021-04500-5

**Published:** 2021-09-06

**Authors:** Kazuhiro Hasegawa, Tamon Kabata, Yoshitomo Kajino, Daisuke Inoue, Jiro Sakamoto, Hiroyuki Tsuchiya

**Affiliations:** 1 Department of Orthopaedic Surgery, Suzu General Hospital, 1-1 Nonoemati, Ishikawa 927-1213 Suzu, Japan; 2 Department of Orthopaedic Surgery, Graduate School of Medical Science, Kanazawa University, 13-1 Takaramachi, Ishikawa 920-8641 Kanazawa, Japan; 3 Bio Engineering Laboratory, School of Mechanical Engineering, Kanazawa University, Kakumamati, Ishikawa 920-1192 Kanazawa, Japan

**Keywords:** Finite element analysis, Osteoarthritis, Pelvic tilt, Acetabular dysplasia

## Abstract

**Background:**

Finite element analysis (FEA) has been previously applied for the biomechanical analysis of acetabular dysplasia and osteotomy. However, until now, there have been little reports on the use of FEA to evaluate the effects of pelvic tilt on stress distribution in the acetabulum.

**Methods:**

We used the Mechanical Finder Ver. 7.0 (RCCM, Inc., Japan) to construct finite element models based on 3D-CT data of patients, and designed dysplasia, borderline, and normal pelvic models. For analysis, body weight was placed on the sacrum and the load of the flexor muscles of the hip joint was placed on the ilium. The pelvic tilt was based on the anterior pelvic plane, and the pelvic tilt angles were -20°, 0°, and 20°. The load of the flexor muscle of the hip joint was calculated using the moment arm equation.

**Results:**

All three models showed the highest values of von Mises stress in the -20° pelvic tilt angle, and the lowest in the 20° angle. Stress distribution concentrated in the load-bearing area. The maximum values of von Mises stress in the borderline at pelvic tilt angles of -20° was 3.5Mpa, and in the dysplasia at pelvic tilt angles of 0° was 3.1Mpa.

**Conclusions:**

The pelvic tilt angle of -20° of the borderline model showed equal maximum values of von Mises stress than the dysplasia model of pelvic tilt angle of 0°, indicating that pelvic retroversion of -20° in borderline is a risk factor for osteoarthritis of the hip joints, similar to dysplasia.

## Background

Osteoarthritis of the hip is the primary disease resulting in total hip arthroplasty (THA).　In Japan, acetabular dysplasia is one of the most important factors associated with osteoarthritis of the hip [1, 2]. Acetabular dysplasia results in decreased acetabular cover of the femoral head and increased pressure on the hip, which generates articular cartilage failure, causing osteoarthritis of the hip. Generally, acetabular dysplasia is evaluated using the central-edge (CE) angle on radiographs (anteroposterior view) [3, 4]. However, osteoarthritis of the hip joint occasionally develops in patients with a normal CE angle. Moreover, pelvic retroversion has been recently shown to result in decreased acetabular cover of the femoral head, causing osteoarthritis of the hip [5].

Finite element analysis (FEA) has been used in the biomechanical analysis of osteotomy, arthroplasty,　joint and the spine area. However, few reports exist using FEA to evaluate the effects of pelvic tilt on stress distribution in the acetabulum [6–12]. Therefore, in the present study, we evaluated the effects of pelvic tilt on stress distribution in the acetabulum using FEA with three-dimensional (3D) computed tomography (CT).

## Methods

**Fig. 1:**
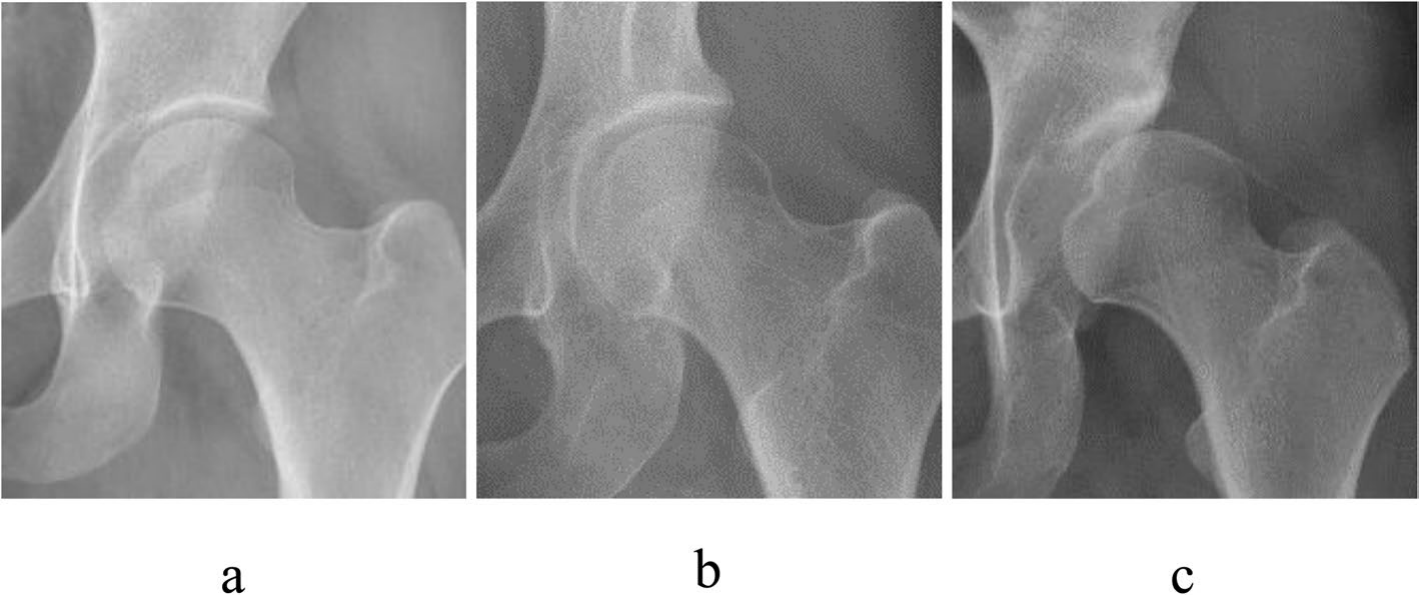
Radiographs (anteroposterior view) of the 3 candidates in present study. **a**. 35 years female who was normal acetabulum (CE angle was 30°). **b**. 39 years female who was borderline acetabular dysplasia (CE angle was 20°). c. 33 years female who was acetabular dysplasia (CE angle was 0°)

**Fig. 2:**
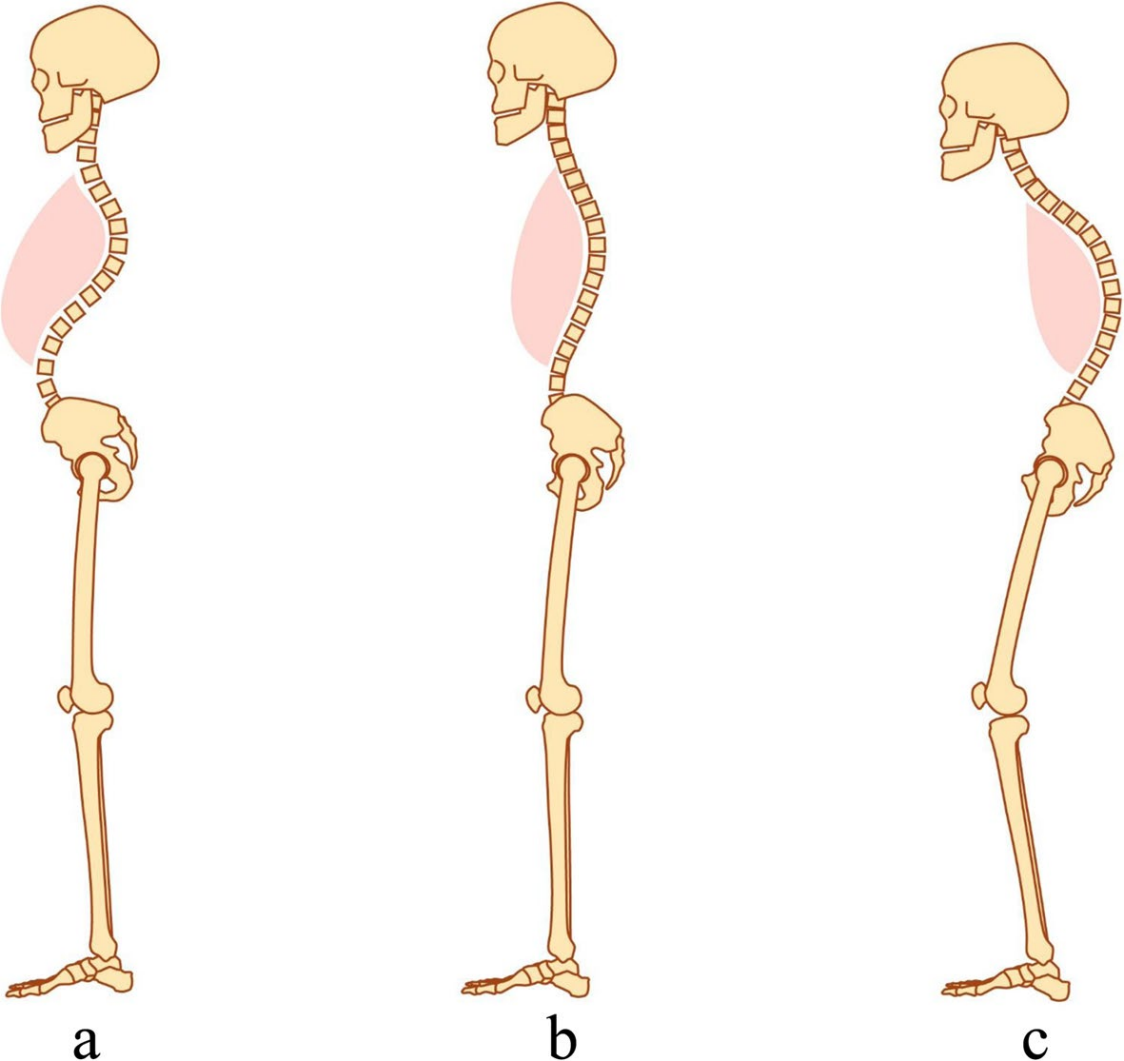
The figures of pelvic postures. **a**. pelvic anteversion posture. **b**. pelvic normal posture. **c**. pelvic retroversion posture

The study utilized DICOM CT images (helical CT scanner, Lightspeed VCT; GE Medical Systems, Milwaukee, WI, USA; 1 mm slice thickness and 2.5 mm pitch) from 3 female patients. Based on the CT data, models were created for normal (35-year-old females; CE angle, 30°), borderline acetabular dysplasia (39-year-old female; CE angle, 20°), and an acetabular dysplasia hip (33-year-old female; CE angle, 0°) (Fig. 1). The rate of the femoral head cover and stress distribution of acetabulum were evaluated. In addition, the influence of the pelvic tilt on acetabular stress distribution was examined for pelvic anteversion, normal, and retroversion postures (Fig. 2). All patients provided informed consent and using of these data was conducted with the approval of our institutional ethical committee.

### Rate of femoral head cover

CT-based templating software (ZedHip; Lexi Co, Tokyo, Japan) was used to measure the rate of femoral head cover [13]. The rate of femoral head cover was assessed at pelvic tilt angles (based on the anterior pelvic plane (APP)) of 20° anteversion, 0°, and 20° retroversion (-20°).

### Stress distribution of acetabulum: FEA

**Fig. 3:**
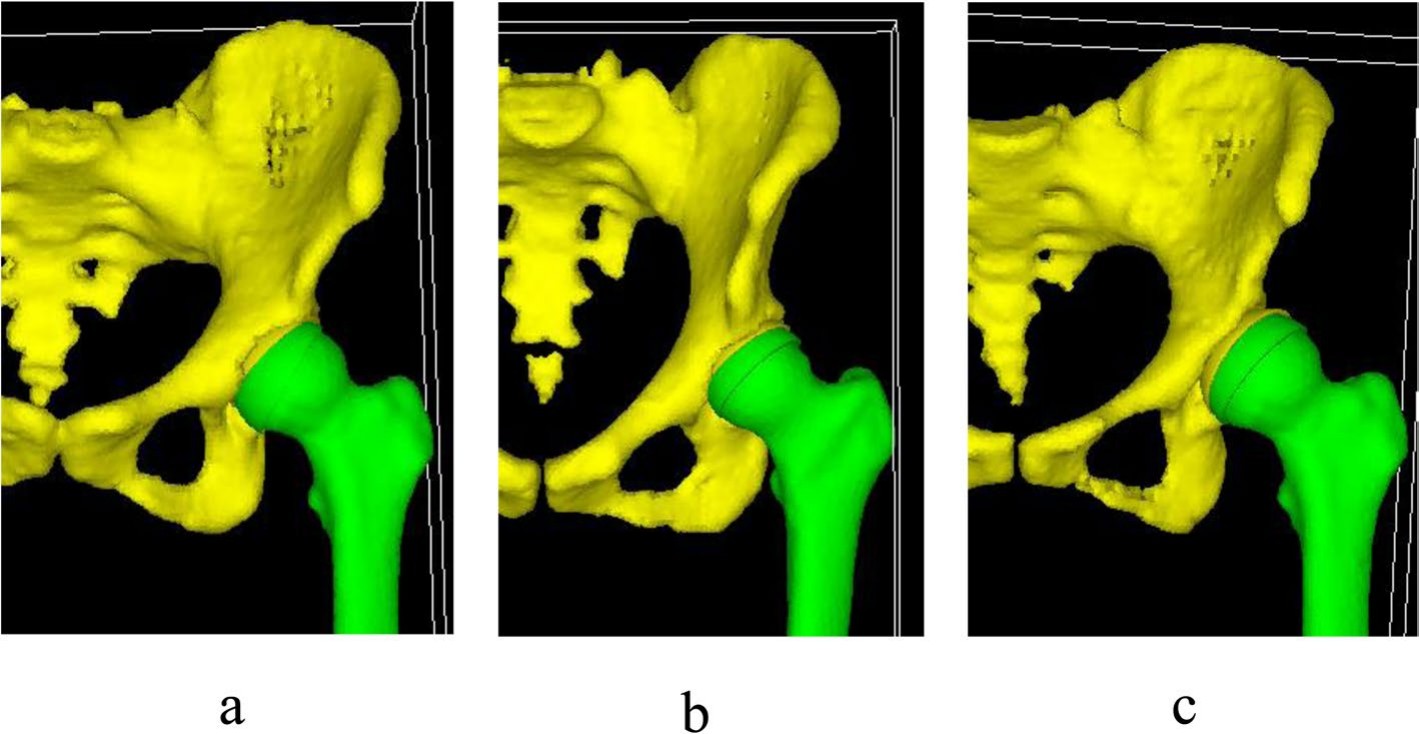
The figures of FEA models. **a**. A model of normal acetabulum. **b**. A model of acetabular borderline dysplasia. **c**. A model of acetabular dysplasia

**Fig. 4:**
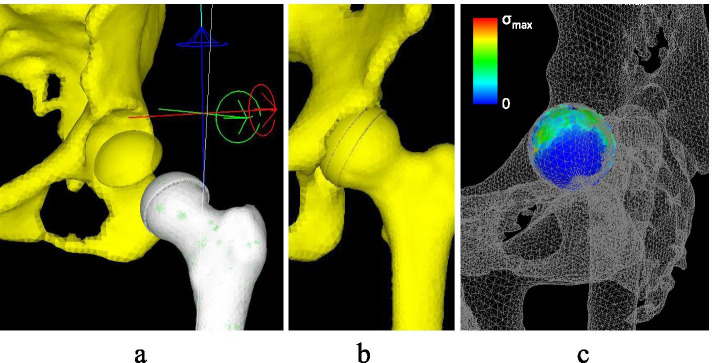
**a**, **b**. The figures of FEA models. A cartilage layer with a mean thickness of approximately 1.5 mm on the femoral head and acetabulum was created using computer-aided design (CAD) data. **c**. the stress distribution on acetabular cartilage was evaluated in all models

**Fig. 5:**
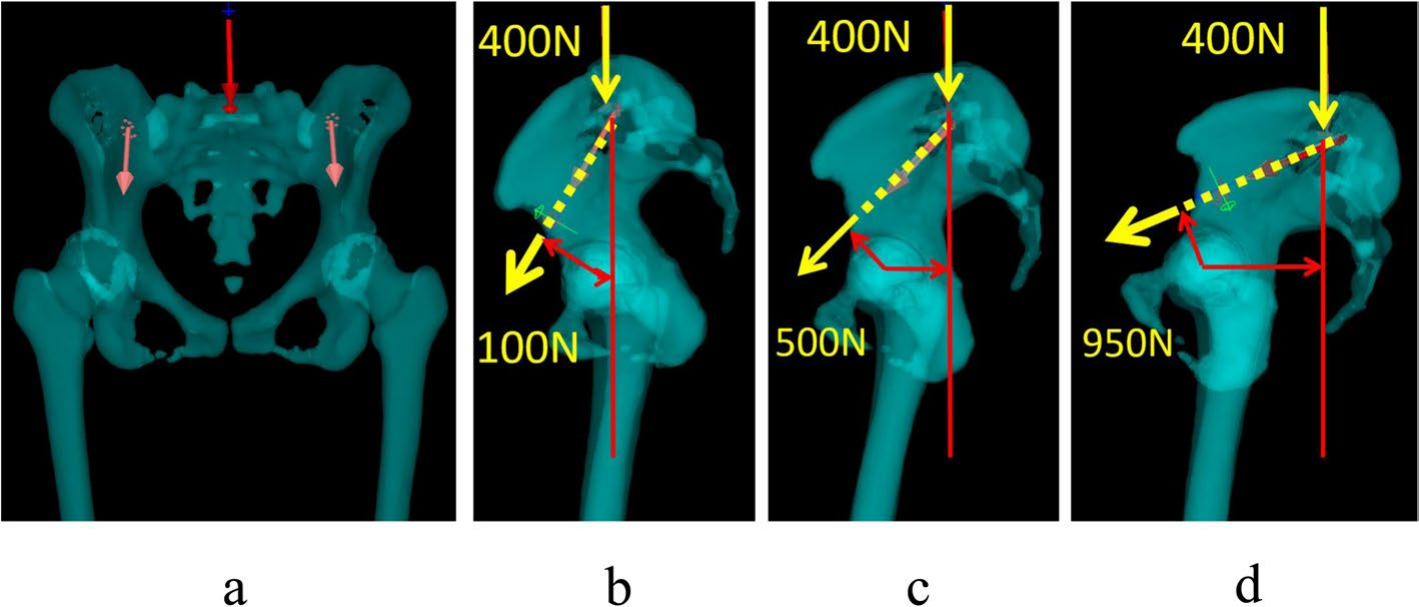
The FEA model that showed body weight was placed on the superior aspect of the sacrum and the load of the flexor muscles of the hip joint was placed on the ilium. **a**. Anteroposterior view of the FEA model. **b**. Lateral view of pelvic 20° anteversion model. The load of the flexor muscle (100 N) of the hip was calculated using the moment arm equation. **c**. Lateral view of pelvic 0 anteversion model. The load of the flexor muscle (500 N) of the hip was calculated using the moment arm equation. **d**. Lateral view of pelvic 20° retroversion model. The load of the flexor muscle (950 N) of the hip was calculated using the moment arm equation

Mechanical Finder Version 7.0 (RCCM Inc., Tokyo, Japan) was used to construct finite element modeling based on the 3D-CT data. Models for normal, borderline dysplasia, and a dysplasia hip (pelvis and femur) were created (Fig. 3). A cartilage layer with a mean thickness of approximately 1.5 mm on the femoral head and acetabulum was created using computer-aided design (CAD) data in all models. And the stress distribution on acetabular cartilage was evaluated (Fig. 4). Four noded linear solid tetrahedral elements were used for the pelvis, femur, and cartilage. Mesh size was 3 mm. The mesh size and elements were set based on previous papers [6, 7]. Briefly, the models were meshed using liner tetrahedral elements with a 3-mm element edge length, which were used in previous study conducted at our institute [6]. And Ike et al. evaluated that the pelvic FEA model created by Mechanical Finder (RCCM, Tokyo, Japan) with 2–4 mm mesh was the finest in sensitivity testing [7]. A total of 9 FEA models were created by adjusting the angle between the pelvis and the femur (normal, borderline dysplasia, and dysplasia models each at pelvic tilt angles based on APP of 20°, 0°, and -20°) (Figs. 3 and 5). The solid was set in Mechanical Finder Version 7.0 (RCCM Inc., Tokyo, Japan). Non-uniform solid was used for the pelvis created from the CT data. Young’s modulus was calculated from the Hounsfield units values of the CT data on the basis of Keyak’s rule to determine the apparent density of each element (Tables 1 and 2) [14]. Uniform solid was used for cartilage and femur imported from CAD data.

**Table 1:** The conversion equation from Hounsfield units to bone density (p; g/cm3)

p = (HU + 1.4246) × 0.001 / 1.0580 : (-1 < HU)
p = 1.0 × 10^-8^ : (HU ≤ -1)
HU: Hounsfield units

**Table 2:** Keyak's fomula

Bone density (p; g/cm^3^)	Young’s modulus (E; MPa)
p = 0	0.001
0 < p ≤ 0.27	33900p^2.20^
0.27 < p ≤ 0.60	5307p + 469
0.60 ≤ p	10200p^2.01^

Keyak's fomula states that the Poisson's ratio is constant at 0.25. Poisson’s ratio for the cartilage was set to 0.45 and femur was set to 0.3. Young’s modulus for the cartilage was 15 MPa and femur was 17,000 MPa. (Table 3) [14, 15]. The normal hip model consisted of 65,098 nodes and 291,469 elements, the borderline hip model consisted of 65,070 nodes and 295,145 elements, and the acetabular dysplasia hip model consisted of 63,070 nodes and 279,611 elements.

**Table 3:** material properties

Material	Young’s Modulus (E; MPa)	Poisson’s ratio
Pelvic bone	Keyak’s formula	0.25
Femoral bone	17,000	0.3
Cartilage	15	0.45

We created FEA models in the standing position with both legs, set the boundary conditions and loading conditions, and performed static analysis. For analysis, the body weight was placed on the superior aspect of the sacrum [15] with a vertical load of 400 N, which was determined by subtracting the weight of the two legs from the body weight (60 kg). The load of the flexor muscles of the hip joint (iliopsoas and rectus femoris) was placed on the ilium and was calculated using the moment-arm equation (Fig. 5). In the boundary conditions, the distal end of the bilateral femur was fixed in all directions. And, the stress distribution on acetabular cartilage was evaluated using the maximum von Mises stress.

## Results

### Rate of femoral head cover

**Fig. 6:**
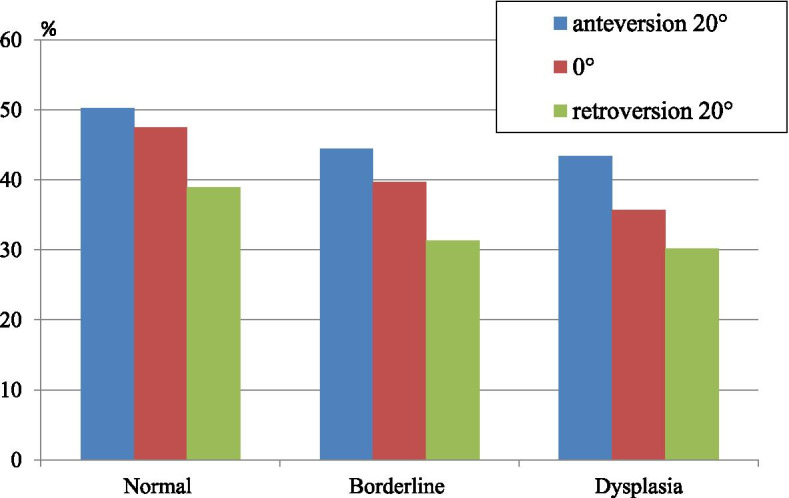
The graph showing the rate of femoral head covers each models

With a pelvic tilt angle of 20°, the rate of femoral head cover in the normal, borderline dysplasia, and dysplasia models was 50.3%, 44.5%, and 43.4%, respectively. With a pelvic tilt angle of 0°, the rate was 47.5%, 39.7%, and 35.7%, respectively. With a pelvic tilt angle of -20°, the rate was 38.9%, 31.4%, and 30.2%, respectively (Fig. 6). Thus, all three models showed the highest rate of femoral head cover with a 20° pelvic tilt angle, and the lowest rate with a -20° angle. Moreover, the borderline dysplasia model with a -20° pelvic tilt angle demonstrated a lower rate of femoral head cover compared to that for the dysplasia model with a pelvic tilt angle of 0°.

### Stress distribution on the acetabulum: FEA

**Fig. 7:**
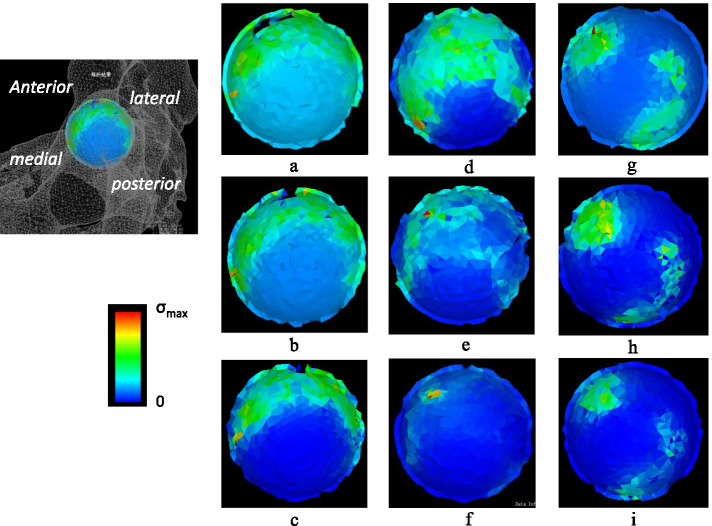
The figure showing stress distribution on acetabular cartilage. **a**. The normal hip at pelvic tilt angles of 20°. **b**. The normal hip at pelvic tilt angles of 0°. **c**. The normal hip at pelvic tilt angles of -20°. **d**. The acetabular borderline dysplasia at pelvic tilt angles of 20°. **e**. The acetabular borderline dysplasia at pelvic tilt angles of 0°. **f**. The acetabular borderline dysplasia at pelvic tilt angles of -20°. **g**. The acetabular dysplasia at pelvic tilt angles of 20°. **h**. The acetabular dysplasia at pelvic tilt angles of 0°. **i**. The acetabular dysplasia at pelvic tilt angles of -20°

**Fig. 8:**
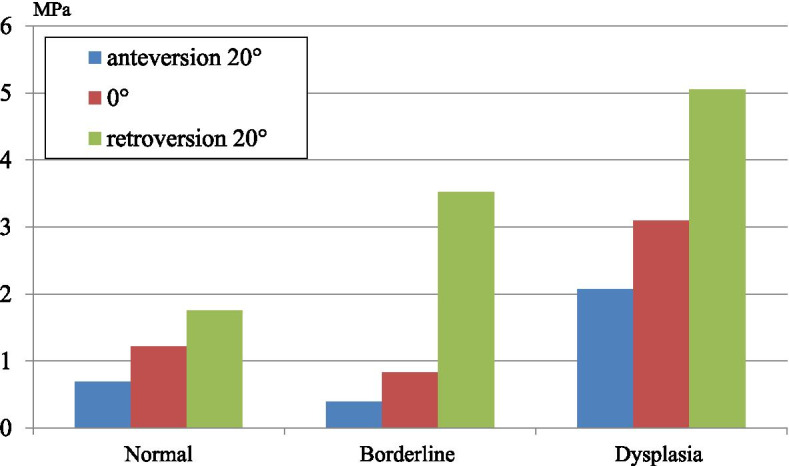
The graph showing maximum von Mises stress value on acetabular cartilage of each FEA models

In the normal model, the stress distribution was widely concentrated around the load-bearing area. When the pelvis was tilted backwards, the stress distribution showed a tendency to focus on the load-bearing area. In the borderline dysplasia model, the stress distribution was concentrated in the anterior and posterior load-bearing area. In addition, the stress distribution was strongly concentrated in the anterior load-bearing area at a pelvic tilt angle of -20°. Similarly, in the dysplasia model, the stress distribution was concentrated in the anterior and posterior load-bearing area, and when the pelvis was tilted backwards, the stress distribution showed a tendency to focus on the anterior load-bearing area. All three models showed the highest von Mises stress value with a -20° pelvic tilt angle, and the lowest stress values with a 20° angle. The borderline dysplasia model with a pelvic tilt angle of -20° had a similar maximum von Mises stress value compared to that for the dysplasia model of pelvic tilt angle of 0° (3.5 Mpa and 3.1 Mpa, respectively) (Figs. 7 and 8).

## Discussion

Recently, pelvic retroversion was reported to result in decreased acetabular cover of the femoral head [16]. In patients with osteoarthritis of the hip, pelvic anteversion is thought to increase the rate of femoral head cover and decrease the load on the acetabulum. Moreover, pelvic retroversion with lumber degenerative kyphosis results in decreased acetabular cover of the femoral head and increased load on the acetabulum [5]. In the present study, models for normal, borderline dysplasia, and dysplasia hips showed the highest von Mises stress in pelvic retroversion, and the lowest in pelvic anteversion. These results demonstrate that pelvic retroversion increases the stress on the acetabulum regardless of pelvic shape.

In the present study, the body weight was placed on the superior aspect of the sacrum based on previous research [15]. By necessity, the load on the flexor muscles of the hip joint was calculated using the moment-arm equation against this body weight to keep the attitude. The body weight for all models was unified to 400 N in order to unify the load conditions across models. Young’s modulus was calculated from the Hounsfield values in the CT data on the basis of Keyak’s rule to determine the apparent density of each element in pelvis as in previous studies [14]. Furthermore, the transmission of the cartilage layer separating the two layers of the acetabular and femoral head sides was designed to closely model the living body.

In the normal hip model, the stress distribution was widely concentrated around the load-bearing area, showing a tendency to focus on the load-bearing area when the pelvis was tilted backwards. In contrast, the stress distribution was concentrated in the anterior and posterior load-bearing area in the borderline dysplasia model, and the stress distribution was strongly concentrated in the anterior load-bearing area at a pelvic tilt angle of -20°. Thus, three-dimensional acetabular dysplasia was involved in addition to the low CE angle for the borderline pelvis [17–19].

Dysplasia results in osteoarthritis of the hip due to decreased acetabular cover of the femoral head [1, 2, 20, 21]. Until now, few reports exist on the use of FEA in evaluating the effects of pelvic tilt on stress distribution in the acetabulum. In the present study, we were able to assess of the influence of pelvic tilt on stress distribution in the acetabulum. The load in the borderline dysplasia model at a pelvic tilt angle of -20° was similar to that for the dysplasia model at a pelvic tilt angle of 0°, which is a risk factor for osteoarthritis. Therefore, borderline dysplasia with a pelvic tilt angle of -20° may also be a risk factor for osteoarthritis of the hip.

The present study has several limitations to discuss. First, the number of the patients was limited due to the involvement of FEA. Second, dysplasia, borderline dysplasia, and a normal pelvis could not be compared in similar patients. Thus, models of different patients were compared. Third, the study did not examine data of a dynamic condition.

## Conclusions

In conclusion, pelvic retroversion resulted in decreased acetabular cover of the femoral head and increased load on the hip joint. A pelvic tilt angle of -20° in borderline dysplasia had a similar maximum von Mises stress value as that for a pelvic tilt angle of 0° in dysplasia, suggesting that a pelvic retroversion of -20° in borderline dysplasia is a risk factor for osteoarthritis of the hip joints.

## Data Availability

The datasets used and/or analysed during the current study are available from the corresponding author on reasonable request.

## Abbreviations

FEA: Finite element analysis
THA: Total hip arthroplasty
CE: Central-edge
APP: Anterior pelvic plane
CAD: Computer-aided design
